# Supplementary material for: The Differential Metabolic Signature of Breast Cancer Cellular Response to Olaparib Treatment
Source: Cancers (Basel). 2022 Jul 27;14(15):3661. doi: 10.3390/cancers14153661 (PMC9367310; doi:10.3390/cancers14153661)
Supplement: Supplementary file 1 [file cancers-14-03661-s001.zip › cancers-1825281-supplementary.pdf]

Supplementary materials

Table S1. Cell lines used in this study and their corresponding clinicopathologic profiles

Cell Line	Histology	Subtype	Immunoprofile	Genetic alterations
**MCF-7**	Metastatic Adenocarcinoma	Luminal A	ER+, PR+, HER2-	PIK3CA, CDKN2A, GATA3, PIK3CA, TP53
**MDA-MB-231**	Metastatic Adenocarcinoma	Basal	ER-, PR-, HER2-	BRAF, CDKN2A, KRAS, NF2, TP53
**HCC1937**	Primary Ductalcarcinoma	Basal-like	ER-, PR-, HER2-	BRCA1, TP53

ER: estrogen receptor, PR: progesterone receptor, and HER2: Human epidermal growth factor 2 receptor).

Elution Gradient used for LC-MS
Buffer A composition:10 mM ammonium acetate in 95% acetonitrile, 5% water with 0.1% acetic acid
Buffer B composition: 10 mM ammonium acetate in 50% acetonitrile, 50% water with 0.1% acetic acid

Table S1. Corresponding elution gradient used for the chromatographic separation of metabolite extracts

Retention (min)	Flow (mL/min)	%A	%B	Curve
0.000	0.500	99.0	1.0	5
1.000	0.500	99.0	1.0	5
3.000	0.500	85.0	15.0	5
6.000	0.500	50.0	50.0	5
9.000	0.500	5.0	95.0	5
10.000	0.500	5.0	95.0	5
10.500	0.500	99.0	1.0	5
14.000	0.500	99.0	1.0	5

Table S3. Normality test for cell viability and immunofluorescence quantification data.

Cell viability	
**Cell line**	**p-value**
**MCF7**	**0.5**
**MDA-MB-231**	**0.3**
**HCC1937**	**0.3**

Immunofluorescence		
**Cell line**	**p-value_p53**	**p-value_ɣH2AX**
**MCF7**	**0.7**	**0.1**
**MDA-MB-231**	**0.3**	**0.9**
**HCC1937**	**0.7**	**0.1**

The Shapiro-Wilk test has been performed with R software. p-values of >0.05 were considered as normally distributed.

LC-MS/MS method summary

- **Method Settings**

Application Mode: Small Molecule

Method duration (min): 14

- **Global Parameters**

Ion Source

Ion Source type: H-ESI
Spray Voltage: Static
Positive Ion (V): 3900
Negative Ion (V): 2700
Gas Mode: Static
Sheath Gas (Arb): 40
Aux Gas (Arb): 10
Sweep Gas (Arb): 1
Ion Transfer Tube Temp (°C): 320
Vaporizer Temp (°C): 300
APPI Lamp: Not in use
Use Ion Source settings from Tune: False
FAIMS Mode: Not Installed

MS Global Settings

Infusion Mode: Liquid Chromatography
Expected LC Peak Width (s): 6
Advanced Peak Determination: False
Mild Trapping: True
Default Charge State: 1
Enable Xcalibur AcquireX method modifications: False
Internal Mass Calibration: EASY-IC™
Mode: Run Start

- **Experiment**

Start Time (min): 0

End Time (min): 14

Full Scan

Orbitrap Resolution: 60,000
Scan Range (m/z): 70-1050

RF Lens (%): 50
AGC Target: Standard
Maximum Injection Time Mode: Custom
Maximum Injection Time (ms): 100
Microscans: 1
Data Type: Profile
Polarity: Positive/Negative
Source Fragmentation: Disabled
Use EASY-C™: On

- Filters

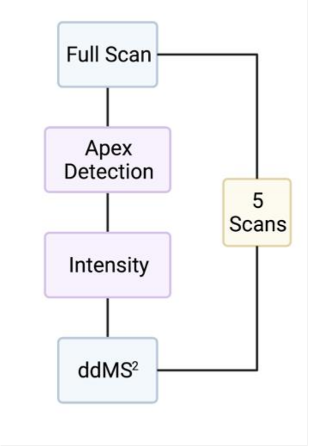

Apex Detection
Desired Apex Window (%): 50
Intensity
Intensity Threshold: 5.0e4
Data Dependent Mode: Number of Scans
Number of Dependent Scans: 5
ddMS^2 Scan
Multiplex Ions: False
Isolation Window (m/z): 2
Isolation Offset: Off
Collision Energy Type: Normalised
HCD Collision Energies (%): 15,30,45
Orbitrap Resolution: 15,000
Scan Range Mode: Auto
AGC Target: Standard
Maximum Injection Time Mode: Auto
Microscans: 1
Data Type: Profile
Use EASY-IC™: On

Mass Spectrometry Data Processing

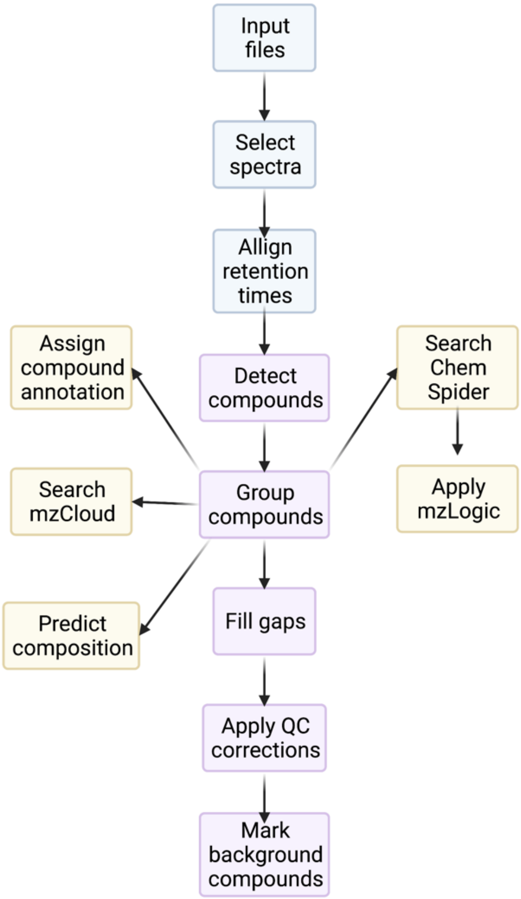

- **Input Files**

.raw data

- **Select Spectra**

Spectrum Properties Filter:

Lower RT Limit: 0
Upper RT Limit: 0
First Scan: 0
Last Scan: 0
Ignore Specified Scans: (not specified)
Lowest Charge State: 0
Highest Charge State: 0
Min. Precursor Mass: 0 Da
Max. Precursor Mass: 5000 Da
Total Intensity Threshold: 0
Minimum Peak Count: 1

Scan Event Filters:

Mass Analyzer: (not specified)
MS Order: Any
Activation Type: (not specified)
Min. Collision Energy: 0
Max. Collision Energy: 1000

Scan Type: Any
Polarity Mode: Is -

Peak Filters:
S/N Threshold (FT-only): 1.5

Replacements for Unrecognized Properties:
Unrecognized Charge Replacements: 1
Unrecognized Mass Analyzer Replacements: ITMS
Unrecognized MS Order Replacements: MS2
Unrecognized Activation Type Replacements: CID
Unrecognized Polarity Replacements: +
Unrecognized MS Resolution@200 Replacements: 60000
Unrecognized MSn Resolution@200 Replacements: 30000

General Settings:
Precursor Selection: Use MS(n - 1) Precursor
Use Isotope Pattern in Precursor Reevaluation: True
Provide Profile Spectra: Automatic
Store Chromatograms: False

- Align Retention Times

General Settings:
Alignment Model: Adaptive curve
Alignment Fallback: None
Maximum Shift [min]: 0.3
Shift Reference File: True
Mass Tolerance: 3 ppm
Remove Outlier: True

- Detect Compounds

General Settings:
Mass Tolerance [ppm]: 3 ppm
Intensity Tolerance [%]: 30
S/N Threshold: 3
Min. Peak Intensity: 500000
Base Ions: [M+H]+1; [M-H]-1
Min. Element Counts: C H
Max. Element Counts: C90 H190 Br3 Cl4 K2 N10 Na2 O15 P6 S5

Peak Detection:
Filter Peaks: True
Max. Peak Width [min]: 0.5
Remove Singlets: True
Min. # Scans per Peak: 5
Min. # Isotopes: 1

Isotope Grouping:
Min. Spectral Distance Score: 0
Remove Potentially False Positive Isotopes: True

- Group Compounds

Compound Consolidation:

Mass Tolerance: 5 ppm
RT Tolerance [min]: 0.2
Fragment Data Selection:
Preferred Ions: [M+H]+1; [M-H]-1

- Fill Gaps

General Settings:

Mass Tolerance: 5 ppm
S/N Threshold: 1.5
Use Real Peak Detection: True

- Apply QC Correction

General Settings:

Regression Model: Linear
Min. QC Coverage [%]: 30
Max. QC Area RSD [%]: 30
Max. Corrected QC Area RSD [%]: 25
Max. # Files Between QC Files: 15

- Mark Background Compounds

General Settings:

Max. Sample/Blank: 5
Max. Blank/Sample: 0
Hide Background: True

- Search ChemSpider

Search Settings:

Database(s): ChEBI; Human Metabolome Database
Search Mode: By Formula or Mass
Mass Tolerance: 5 ppm
Max. # of results per compound: 100
Max. # of Predicted Compositions to be searched per Compound: 3
Result Order (for Max. # of results per compound): Order By Reference Count (DESC)
Predicted Composition Annotation:
Check All Predicted Compositions: False

- Apply mzLogic

Search Settings:

FT Fragment Mass Tolerance: 10 ppm
IT Fragment Mass Tolerance: 0.4 Da
Max. # Compounds: 0
Max. # mzCloud Similarity Results to consider per Compound: 10
Match Factor Threshold: 30

- Predict Compositions

Prediction Settings:

Mass Tolerance: 5 ppm
Min. Element Counts: C H
Max. Element Counts: C90 H190 Br3 Cl4 N10 O18 P3 S5
Min. RDBE: 0
Max. RDBE: 40
Min. H/C: 0.1
Max. H/C: 4
Max. # Candidates: 10
Max. # Internal Candidates: 200

Pattern Matching:

Intensity Tolerance [%]: 30
Intensity Threshold [%]: 0.1
S/N Threshold: 3
Min. Spectral Fit [%]: 30
Min. Pattern Cov. [%]: 90
Use Dynamic Recalibration: True

Fragments Matching:

Use Fragments Matching: True
Mass Tolerance: 5 ppm
S/N Threshold: 3

- Assign Compound Annotations

General Settings:

Mass Tolerance: 5 ppm
Data Sources:
Data Source #1: mzCloud Search
Data Source #2: Predicted Compositions
Data Source #3: MassList Search
Data Source #4: ChemSpider Search
Data Source #5: Metabolika Search
Data Source #6: (not specified)
Data Source #7: (not specified)

Scoring Rules:

Use mzLogic: True
Use Spectral Distance: True
SFit Threshold: 20
SFit Range: 20

- Search mzCloud

General Settings:

Compound Classes: All
Precursor Mass Tolerance: 10 ppm
FT Fragment Mass Tolerance: 10 ppm
IT Fragment Mass Tolerance: 0.4 Da
Library: Autoprocessed; Reference

Post Processing: Recalibrated
Max. # Results: 10
Annotate Matching Fragments: True

DDA Search:
Identity Search: Cosine
Match Activation Type: True
Match Activation Energy: Match with Tolerance
Activation Energy Tolerance: 20
Apply Intensity Threshold: True
Similarity Search: None
Match Factor Threshold: 60

DIA Search:
Use DIA Scans for Search: False
Max. Isolation Width [Da]: 500
Match Activation Type: False
Match Activation Energy: Any
Activation Energy Tolerance: 100
Apply Intensity Threshold: False
Match Factor Threshold: 20

- Differential Analysis

General Settings:
Log10 Transform Values: True

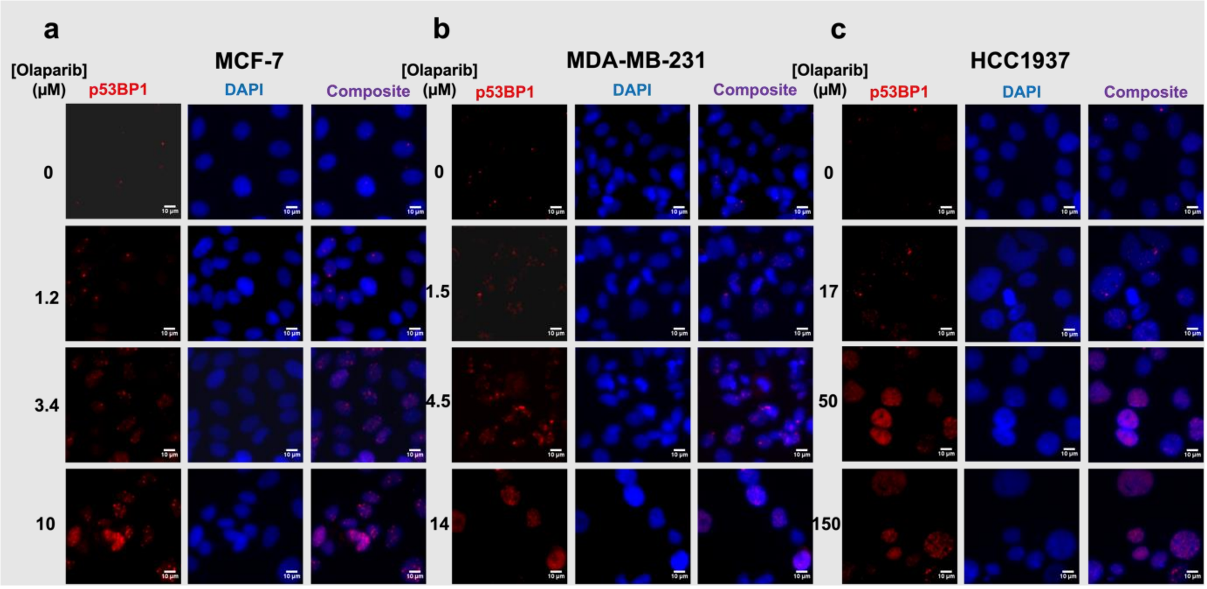

Figure S1. The formation of p53BP1 foci in response to treatment with either growth medium or medium containing olaparib at various doses. Representative images of immunolabelled P53BP1 foci (red), DAPI (blue) nuclear counterstain and composite (p53BP1 (red) and DAPI (blue)) in MCF-7, MDA-MB-231, and HCC1937 cells treated with olaparib for seven days (a-c).

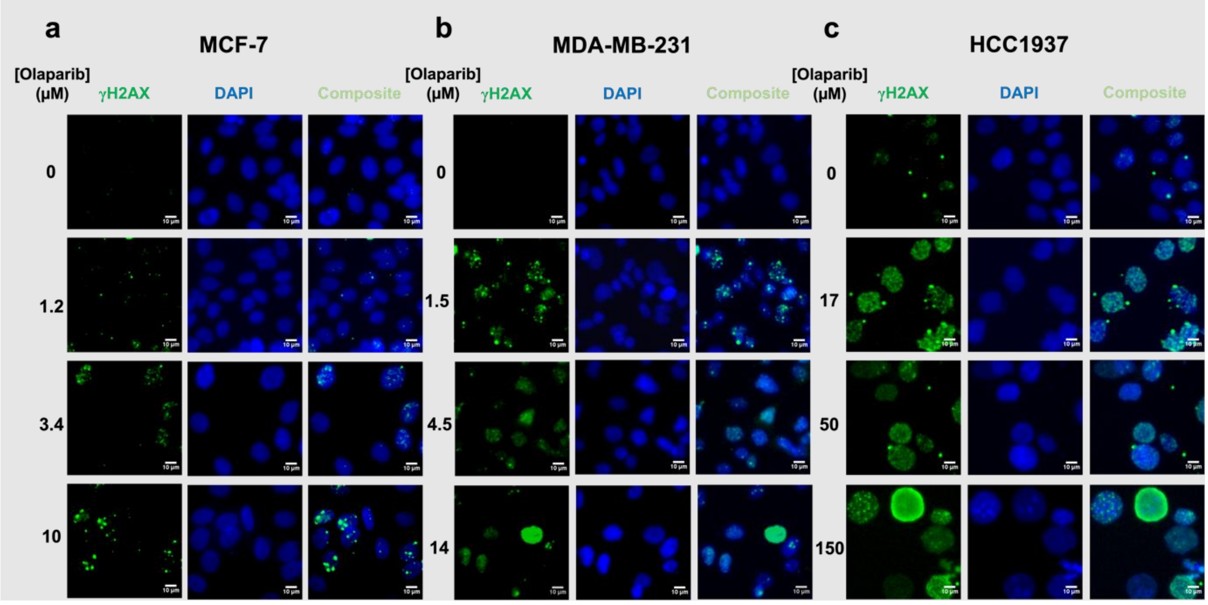

Figure S2. The formation of γH2AX foci formation in response to treatment with either growth medium or medium containing olaparib at various doses. Representative images of immunolabelled γH2AX foci (green), DAPI (blue) nuclear counterstain and composite (γH2AX and DAPI) in MCF-7, MDA-MB-231, and HCC1937 cells treated with for seven days (a-c).

Table S4. ANOVA analysis of olaparib dose-dependent DNA DSB immunofoci formation

Cell line	Foci	p-value
MCF-7	*53BP1*	0.011
	γH2AX	4.876×10^{-10}
MDA-MB-231	*53BP1*	0.0009
	γH2AX	4.096×10^{-10}
HCC1937	*53BP1*	1.204×10^{-6}
	γH2AX	1.441×10^{-5}

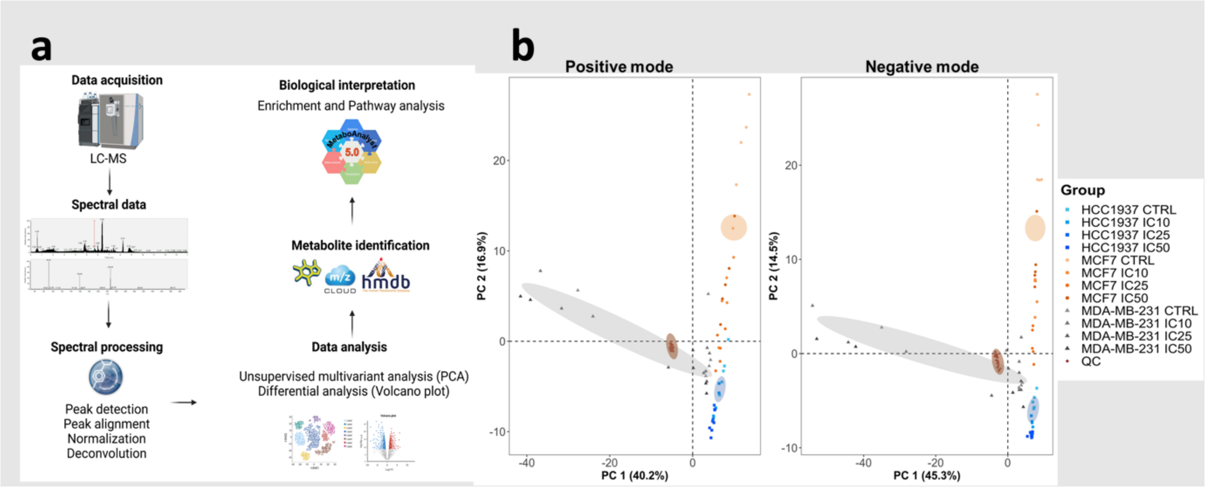

Figure S3. Untargeted mass spectrometry-based metabolomics and data analysis pipeline (a-created in Biorender.com). Global metabolic features identified in MCF7, MDA-MB-231 and HCC1937 upon exposure to IC_{10}, IC_{25} and IC_{50} olaparib doses for seven days acquired in positive and negative ionization modes (b). For each treatment group, five replicates were used. Data points in the two-dimensional PCA score plot were central scaled (right).

Panel of individual PCA pairwise analysis of MCF7, MDA-MB-231 and HCC1937 at their relative IC_{10}, IC_{25} and IC_{50} doses of Olaparib

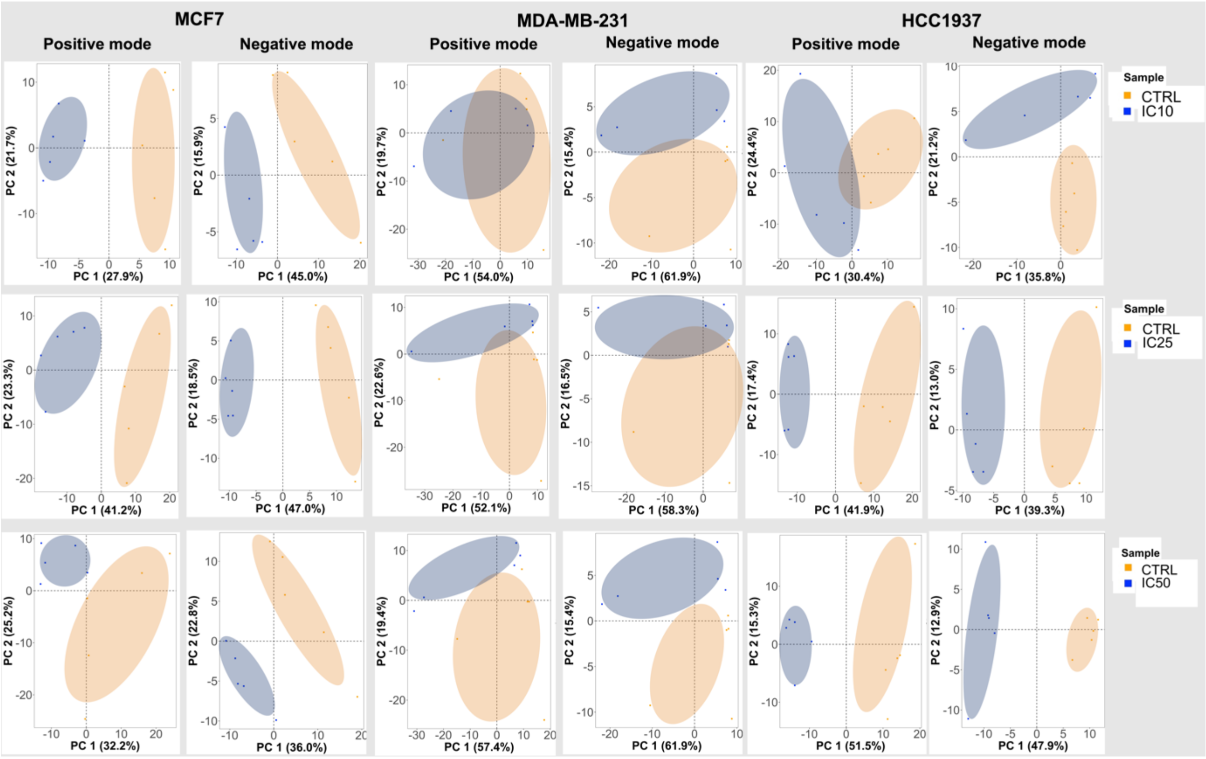

Figure S4. PCA pairwise analysis of untargeted metabolomics data collected, in both positive and negative mode, from MCF7, MDA-MB-231, and HCC1937 cells treated with IC_{10}, IC_{25} and IC_{50} olaparib treatment doses. Data points in the two-dimensional PCA score plot were central scaled. The plot was designed on R through the ggplot2 graphical package (n = 5).

Pairwise differential analysis of metabolites identified in MCF7, MDA-MB-231 and HCC1937 cells in positive and negative mode

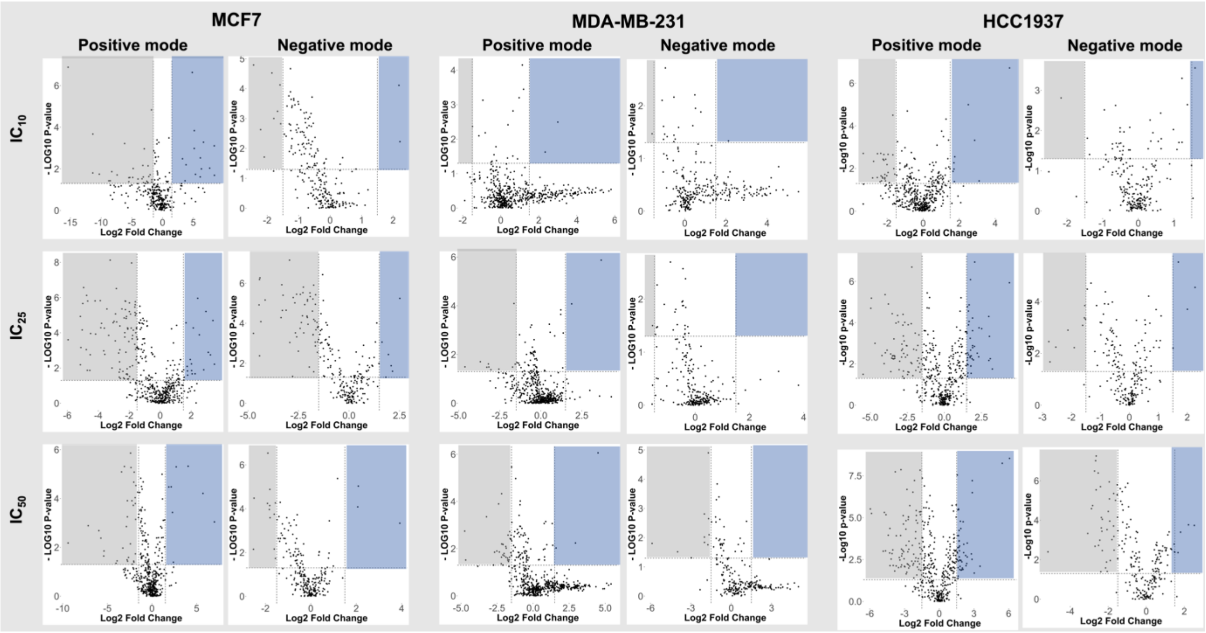

Figure S5. Volcano plots showing the log2 fold change and the -log10 adjusted p-values in metabolite levels induced by treatment with different doses of Olaparib (IC_{10}, IC_{25}, and IC_{50}) in MCF7, MDA-MB-231 and HCC1937 cells. Data were selected at the cut off values adjusted $p < 0.05$ and fold change > 1.5. Blue and grey boxes indicate metabolites significantly enriched and depleted in the different samples, respectively.

Table S5. Global differential number of altered metabolites for samples treated with IC_{10}, IC25 and IC_{50} of Olaparib and their relative control (non-treated) samples.

Sample	HESI +	HESI -
MCF7 IC10/Ctrl	41	10
MCF7 IC25/Ctrl	111	62
MCF7 IC50/Ctrl	41	15
MDA231 IC10/Ctrl	2	1
MDA231 IC25/Ctrl	12	1
MDA231 IC50/Ctrl	34	9
HCC1937 IC10/Ctrl	36	2
HCC1937 IC25/Ctrl	107	13
HCC1937 IC50/Ctrl	134	43

Metabolites identified in both positive and negative mode with *p*-value = < 0.05 and Log2 Fold Change ≥ 1.5.

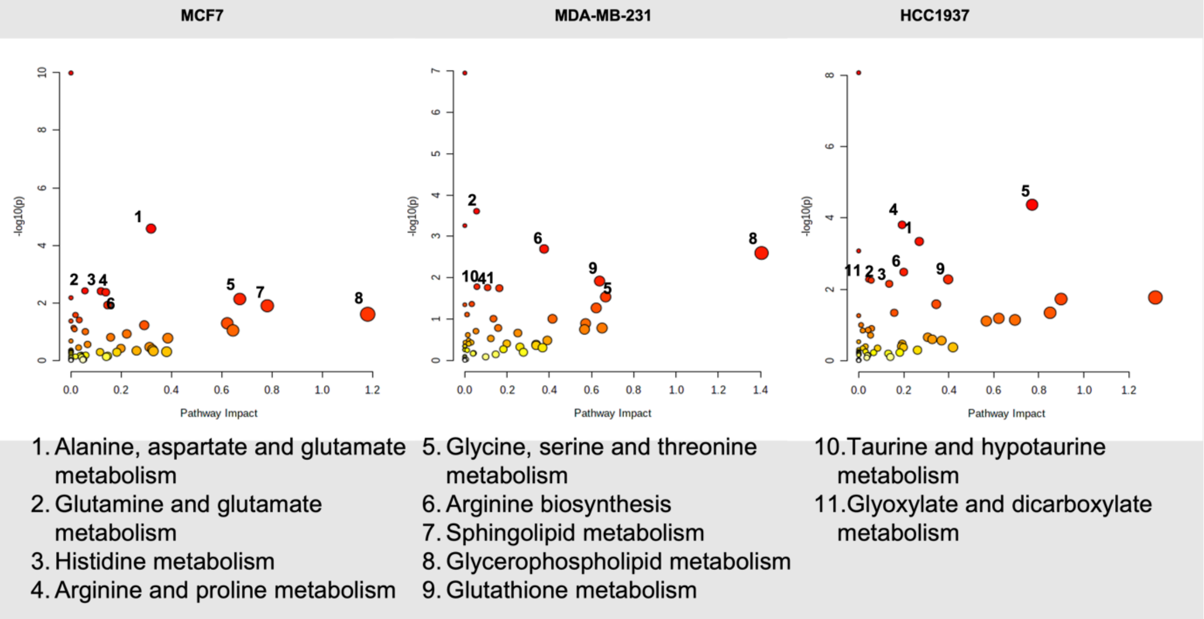

Figure S6. Enrichment analysis of non-treated MCF7, MDA-MB-231 and HCC1937 cells.

Table S6. Metabolic pathways in different breast cancer cells (MCF7, MDA-MB-231, and HCC1937) before and after treatment with IC_{50} dose of Olaparib.

	**- Olaparib**				**+ Olaparib**			
**MCF7**	**Pathways**	**P-value**	**FDR**	**Impact**	**Pathways**	**P-value**	**FDR**	**Impact**
	Aminoacyl-tRNA biosynthesis	1.02E-10	8.57E-09	0	Aminoacyl-tRNA biosynthesis	8.19E-10	6.88E-08	0
	Alanine, aspartate and glutamate metabolism	2.63E-05	0.001104	0.31879	Arginine biosynthesis	4.37E-06	0.000184	0.54769
	Glutamine and glutamate metabolism	3.83E-03	0.072247	0.055556	Alanine, aspartate and glutamate metabolism	1.31E-05	0.000366	0.25749
	Histidine metabolism	3.94E-03	0.072247	0.11935	Arginine and proline metabolism	0.000141	0.002965	0.20848
	Arginine and proline metabolism	4.30E-03	0.072247	0.13859	Glutamine and glutamate metabolism	0.000363	0.006102	0.055556
	Valine, leucine and isoleucine biosynthesis	0.006688	0.088594	0	Valine, leucine and isoleucine biosynthesis	0.000807	0.011293	0
	Glycine, serine and threonine metabolism	0.007382	0.088594	0.67221	Taurine and hypotaurine metabolism	0.002633	0.0316	0.085714
	Arginine biosynthesis	0.012025	0.11847	0.14615	Glycine, serine and threonine metabolism	0.003952	0.040556	0.67221
	Sphingolipid metabolism	0.012694	0.11847	0.78164	Butanoate metabolism	0.004345	0.040556	0.12169
	Glycerophospholipid metabolism	0.025021	0.20274	1.1815	Vitamin B6 metabolism	0.00747	0.062741	0.96579
**MDA-MB-231**	**Pathways**	**P-value**	**FDR**	**Impact**	**Pathways**	**P-value**	**FDR**	**Impact**
	Aminoacyl-tRNA biosynthesis	1.11E-07	9.31E-06	0	Aminoacyl-tRNA biosynthesis	4.87E-08	4.09E-06	0
	Glutamine and glutamate metabolism	0.000246	0.010315	0.055556	Arginine biosynthesis	4.44E-05	0.001297	0.54769
	Valine, leucine and isoleucine biosynthesis	0.000549	0.015362	0	Valine, leucine and isoleucine biosynthesis	4.63E-05	0.001297	0
	Arginine biosynthesis	0.00201	0.042201	0.37538	Glutamine and glutamate metabolism	0.000348	0.007318	0.055556
	Glycerophospholipid metabolism	0.002519	0.042319	1.4051	Arginine and proline metabolism	0.002382	0.040027	0.19139
	Glutathione metabolism	0.012023	0.16708	0.63816	Glycine, serine and threonine metabolism	0.003697	0.05176	0.35674
	Taurine and hypotaurine metabolism	0.016455	0.16708	0.057143	Alanine, aspartate and glutamate metabolism	0.007553	0.087816	0.25155
	Arginine and proline metabolism	0.017286	0.16708	0.10783	Pantothenate and CoA biosynthesis	0.008364	0.087816	0.025568
	Alanine, aspartate and glutamate metabolism	0.017902	0.16708	0.16342	Cysteine and methionine metabolism	0.016897	0.1577	0.74161
	Glycine, serine and threonine metabolism	0.029087	0.24434	0.66667	Taurine and hypotaurine metabolism	0.020936	0.17277	0.057143
**HCC1937**	**Pathways**	**P-value**	**FDR**	**Impact**	**Pathways**	**P-value**	**FDR**	**Impact**
	Aminoacyl-tRNA biosynthesis	8.36E-09	7.03E-07	0	Aminoacyl-tRNA biosynthesis	6.05E-09	5.08E-07	0
	Glycine, serine and threonine metabolism	4.24E-05	0.001781	0.77013	Arginine biosynthesis	4.03E-06	0.000169	0.54769
	Arginine and proline metabolism	0.000156	0.004357	0.19275	Alanine, aspartate and glutamate metabolism	7.18E-05	0.002011	0.25692
	Alanine, aspartate and glutamate metabolism	0.000455	0.009555	0.26907	Glutamine and glutamate metabolism	0.000348	0.007318	0.055556
	Valine, leucine and isoleucine biosynthesis	0.00084	0.014109	0	Arginine and proline metabolism	0.000585	0.009829	0.19275
	Arginine biosynthesis	0.003289	0.046045	0.2	Valine, leucine and isoleucine biosynthesis	0.000774	0.010591	0
	Glyoxylate and dicarboxylate metabolism	0.005277	0.052485	0.043771	Glycine, serine and threonine metabolism	0.000883	0.010591	0.37754
	Glutathione metabolism	0.005277	0.052485	0.39739	Taurine and hypotaurine metabolism	0.002532	0.02658	0.085714
	Glutamine and glutamate metabolism	0.005623	0.052485	0.055556	Cysteine and methionine metabolism	0.004844	0.045211	0.67619
	Histidine metabolism	0.007034	0.05908	0.13548	Butanoate metabolism	0.022626	0.19004	0.12169

FDR = False Discovery Rate.

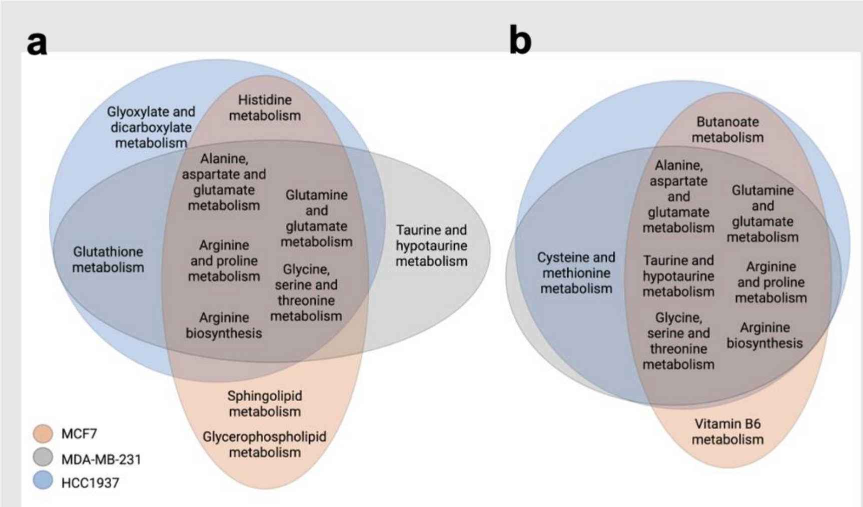

Figure S7. Venn diagram representing the metabolic pathways in MCF7, MDA-MB-231 and HCC1937 cells .a) Baseline metabolic pathways and b) following a seven day treatment with olaparib at IC_{50} doses.

Table S7. Classification of the metabolites identified in MCF7, MDA-MB-231 and HCC1937 at all Olaparib doses (IC$_{10}$, IC$_{25}$ and IC$_{50}$) after seven days treatment.

		**MCF7**						**MDA-MB-231**						**HCC1937**					
**Class**	**Name**	**Log$_2$ fold change IC10**	***p***	**Log$_2$ fold change IC25**	***p***	**Log$_2$ fold change IC50**	***p***	**Log$_2$ fold change IC10**	***p***	**Log$_2$ fold change IC25**	***p***	**Log$_2$ fold change IC50**	***p***	**Log$_2$ fold change IC10**	***p***	**Log$_2$ fold change IC25**	***p***	**Log$_2$ fold change IC50**	***p***
**Amide**	**Nicotinamide**	-0.54	*0.08*	-0.54	*0.26*	-0.14	*0.29*	-0.07	*0.50*	-0.16	*0.05*	-0.45	*0.03*	0.35	0.07	0.33	0.01	-0.1	0.28
**Amine**	**N-Oleoyl ethanolamine**	-1.48	*0.13*	-0.81	*0.63*	-0.08	*0.61*	-0.46	*0.29*	-3.19	*0.02*	-0.61	*0.26*	-1.64	0.17	-2.48	0.03	-1.16	0.02
**Amine**	**Triethanolamine**	1.03	*0.56*	-0.6	*0.03*	0.05	*0.15*	2.87	*0.43*	0.57	*0.76*	3.05	*0.41*	-0.46	0.47	0.42	0.13	0.39	0.50
**Amino acid**	**3-Sulfinoalanine**	-1.05	*0.45*	-1.75	*0.03*	-1.07	*0.15*	-	-	-0.65	*0.33*	-	-	0.32	0.71	-0.41	0.64	-0.67	0.02
**Amino acid**	**4-Guanidinobutanoic acid**	-1.26	*0.01*	-2.03	*0.00*	-1.13	*0.00*	-0.22	*0.49*	-0.28	*0.57*	-0.46	*0.16*	-1.03	0.08	-1.82	0.01	-1.81	0.00
**Amino acid**	**4-Hydroxyproline**	-1.01	*0.00*	-2.1	*0.00*	-1.08	*0.01*	-0.37	*0.16*	-	-	-0.39	*0.14*	-0.33	0.44	-0.3	0.33	-0.87	0.02
**Amino acid**	**4-Oxoproline**	-0.7	*0.34*	0.88	*0.03*	0.16	*0.24*	0.36	*0.55*	-0.27	*0.03*	0.81	*0.06*	0.64	0.03	1.03	0.01	1.47	0.00
**Amino acid**	**Betaine**	-	-	-0.84	*0.00*	-0.04	*0.72*	-0.02	*0.26*	-0.17	*0.11*	-0.44	*0.02*	-0.05	0.72	-0.03	0.49	-0.39	0.00
**Amino acid**	**Choline**	-3.71	*0.11*	-2.14	*0.02*	-2.11	*0.07*	0.06	*0.46*	-1.3	*0.03*	-1.53	*0.00*	-2.08	0.00	-2.87	0.00	-3.71	0.00
**Amino acid**	**Citrulline**	-0.27	*0.58*	1.76	*0.01*	-0.39	*0.04*	-0.33	*0.22*	-0.25	*0.05*	-0.29	*0.02*	-0.08	0.47	0.31	0.51	0.06	0.91
**Amino acid**	**Creatine**	-0.31	*0.57*	-1.68	*0.00*	-0.47	*0.05*	-0.82	*0.01*	-0.35	*0.18*	-1.12	*0.01*	0.05	0.26	-0.37	0.03	-0.7	0.01
**Amino acid**	**Creatinine**	-	-	-0.12	*0.18*	0.1	*0.73*	-0.46	*0.04*	-0.38	*0.06*	-0.36	*0.15*	-0.13	0.44	0.31	0.37	-0.2	0.28
**Amino acid**	**Gamma-Aminobutyric acid**	-0.93	*0.00*	-1.75	*0.00*	-0.74	*0.00*	-0.16	*0.14*	-0.09	*0.15*	-0.2	*0.14*	-0.21	0.71	0.02	0.87	-0.52	0.00
**Amino acid**	**Glycine**	-1.26	*0.00*	-2.08	*0.00*	-0.99	*0.00*	-	-	-	-	-	-	-	-	-	-	-	-

**Amino acid**	**Hypotaurine**	0.29	*0.30*	-0.35	*0.10*	-0.61	*0.03*	0.25	*0.55*	-0.2	*0.07*	-0.54	*0.00*	-0.72	0.02	-0.55	0.00	-1.26	0.00
**Amino acid**	**L-Alanine**	-0.83	*0.00*	-1.66	*0.00*	-0.91	*0.01*	-	-	-	-	-	-	-	-	-	-	-	-
**Amino acid**	**L-Arginine**	6.65	*0.00*	-0.46	*0.02*	-0.07	*0.56*	-0.08	*0.56*	-0.14	*0.65*	0.25	*0.36*	0.02	0.49	0.25	0.06	-0.19	0.08
**Amino acid**	**L-Asparagine**	0.3	*0.33*	-0.38	*0.22*	0.99	*0.04*	-	-	-	-	-	-	0.9	0.16	1.07	0.13	1.31	0.13
**Amino acid**	**L-Aspartic acid**	-0.64	*0.04*	-2.2	*0.00*	-1.38	*0.00*	0.32	*0.07*	0.33	*0.27*	0.79	*0.02*	-0.14	0.89	-0.5	0.01	-1.62	0.00
**Amino acid**	**L-Cystathionine**	5.15	*0.00*	-2.06	*0.00*	-0.32	*0.12*	-0.13	*0.93*	1.19	*0.42*	0.79	*0.64*	-1.68	0.00	-2.2	0.00	-3.8	0.00
**Amino acid**	**L-Glutamic acid**	-1.26	*0.00*	-2.08	*0.00*	-1.3	*0.00*	-0.35	*0.53*	-0.71	*0.02*	-1.14	*0.00*	-0.4	0.11	-0.49	0.03	-2.06	0.00
**Amino acid**	**L-Glutamine**	-11.37	*0.02*	-0.97	*0.00*	-0.38	*0.06*	-0.05	*0.20*	0.17	*0.43*	-0.09	*0.71*	-0.21	0.03	-0.32	0.00	-0.51	0.00
**Amino acid**	**L-Isoleucine**	-	-	-1.49	*0.27*	-0.59	*0.97*	-1.39	*0.98*	1.52	*0.31*	-0.28	*0.65*	0.07	0.18	-0.56	0.73	0.12	0.95
**Amino acid**	**L-Leucine**	-	-	-1.39	*0.00*	-0.41	*0.12*	0.22	*0.40*	-	-	-0.5	*0.07*	-	-	-0.63	0.04	-1.31	0.00
**Amino acid**	**L-Lysine**	-1.11	*0.01*	-1.04	*0.02*	-0.42	*0.34*	-0.3	*0.07*	-0.41	*0.12*	0.09	*0.47*	0.18	0.25	0.28	0.01	-0.35	0.00
**Amino acid**	**L-Methionine**	-0.93	*0.00*	-2.25	*0.00*	-1.04	*0.00*	-0.07	*0.23*	-0.31	*0.03*	-0.39	*0.11*	0.06	0.96	-0.14	0.23	-0.87	0.00
**Amino acid**	**L-Ornithine**	-	-	-	-	-	-	-	-	-	-	-	-	0.18	0.55	0.29	0.08	-0.12	0.66
**Amino acid**	**L-Phenylalanine**	-1.31	*0.74*	-	-	-	-	-0.12	*0.36*	-0.37	*0.03*	-0.28	*0.05*	-0.18	0.86	-0.32	0.19	-0.72	0.00
**Amino acid**	**L-Proline**	-1.26	*0.00*	2.04	*0.12*	-0.33	*0.53*	0.07	*0.64*	-0.25	*0.44*	-0.39	*0.14*	-	-	-	-	-	-
**Amino acid**	**L-Serine**	2.18	*0.00*	2.52	*0.00*	2.09	*0.00*	0.25	*0.23*	0.33	*0.45*	0.88	*0.00*	-0.09	0.69	-0.33	0.01	-0.97	0.00
**Amino acid**	**L-Tryptophan**	-1.57	*0.00*	-2.36	*0.00*	-1.34	*0.00*	-0.33	*0.07*	-0.77	*0.03*	-0.91	*0.01*	-0.27	0.48	-0.75	0.02	-0.84	0.00
**Amino acid**	**L-Tyrosine**	0.08	*0.71*	-2.29	*0.00*	-1.25	*0.00*	-0.35	*0.03*	-0.51	*0.00*	-0.55	*0.02*	0.11	0.02	-0.36	0.13	-1	0.01
**Amino acid**	**L-Valine**	-1.16	*0.00*	-1.41	*0.00*	-0.56	*0.01*	0.12	*0.58*	0.09	*0.91*	0.36	*0.96*	0.07	0.84	0.21	0.97	0.58	0.40
**Amino acid**	**N-a-Acetyl-L-arginine**	-	-	-1.61	*0.00*	-0.64	*0.01*	0.49	*0.52*	-0.19	*0.36*	-0.55	*0.13*	-0.34	0.22	0	0.50	-0.39	0.02

**Amino acid**	**N-Acetylaspartyl glutamic acid**	-0.53	0.11	-1.53	0.00	-0.68	0.07	0.2	0.68	-0.27	0.39	-0.06	0.19	-1.29	0.01	-0.83	0.00	-2.86	0.00
**Amino acid**	**N-Acetylisoleucine**	-0.13	0.02	0.25	0.09	-0.05	0.52	2.42	0.36	0.37	0.77	1.37	0.49	0.02	0.64	0.13	0.02	0.11	0.20
**Amino acid**	**N-Acetylleucine**	-	-	0.87	0.65	0.44	0.74	-	-	0.43	0.59	-	-	-	-	-	-	0.7	0.45
**Amino acid**	**N-Acetyltaurine**	-0.37	0.05	-0.82	0.00	-0.65	0.01	0.14	0.91	-0.16	0.22	-5.89	0.02	-0.74	0.13	-0.7	0.01	-1.08	0.00
**Amino acid**	**Ornithine**	4.83	0.00	-0.4	0.25	-0.23	0.22	-0.54	0.08	-0.61	0.01	-0.26	0.19	0.18	0.55	0.29	0.08	-0.12	0.66
**Amino acid**	**Pyroglutamic acid**	-10.3	0.02	-0.73	0.03	-0.17	0.51	-0.05	0.20	0.19	0.42	-0.29	0.56	-0.25	0.01	-0.34	0.01	-0.61	0.00
**Amino acid**	**Taurine**	-0.37	0.05	-0.82	0.00	-0.65	0.01	0.14	0.91	-0.16	0.22	-5.89	0.02	-0.74	0.13	-0.7	0.01	-1.08	0.00
**Amino acid**	**Thiamine**	5.36	0.38	-1.22	0.00	-0.87	0.00	-1.15	0.01	-0.44	0.05	-0.31	0.25	-0.3	0.47	-0.15	0.43	-1.07	0.00
**Amino acid**	**Threonine**	0.06	0.36	1.4	0.50	0.57	0.94	-1.9	0.60	-0.52	0.09	0.64	0.51	-0.22	0.66	-0.3	0.08	0.67	0.34
**Benzenoid**	**Benzoic acid**	0.64	0.86	0.3	0.43	0.11	0.43	0.39	0.02	0.13	0.56	0.34	0.39	-0.17	0.25	-0.24	0.04	0.76	0.01
**Carbohydrate**	**D-Glucose**	-0.23	0.94	0.96	0.02	0.21	0.23	-0.21	0.38	-0.37	0.04	0.09	0.17	1.09	0.03	0.92	0.00	0.84	0.00
**Carbohydrate**	**Glyceraldehyde 3-phosphate**	-1.45	0.00	-2.31	0.00	-1.43	0.00	0.22	0.27	-0.22	0.18	-1.31	0.02	0.19	0.86	-0.26	0.28	-1.7	0.00
**Carbohydrate**	**Mannose 6-phosphate**	-0.32	0.20	-0.72	0.09	-0.2	0.30	0.78	0.01	0.01	0.87	0.22	0.31	0.33	0.01	0.67	0.00	0.47	0.02
**Carbohydrate**	**N-Acetyl-glucosamine 1-phosphate**	-1.31	0.00	-2.41	0.00	-1.56	0.00	-0.14	0.09	-0.63	0.00	-1.21	0.00	-	-	-	-	-	-
**Carbohydrate**	**Threonic acid**	-0.73	0.03	-0.91	0.01	-0.3	0.68	-0.53	0.04	-0.33	0.11	-0.78	0.02	-0.51	0.01	-0.59	0.00	-1.62	0.00
**Carboxylic acid**	**5-L-Glutamyl-taurine**	2.22	0.01	1.24	0.01	2.07	0.00	-	-	-	-	-	-	-	-	-	-	-	-
**Carboxylic acid**	**Citric acid**	-0.43	0.08	-1.27	0.00	-0.3	0.20	0.1	0.81	-0.23	0.94	-	-	-	-	-	-	-0.91	0.00
**Carboxylic acid**	**Dodecanedioic acid**	0.42	0.70	0.65	0.06	0.47	0.09	1.82	0.40	0.07	0.66	-1.08	0.63	0.25	0.33	0.28	0.15	0.43	0.11

**Carboxylic acid**	**Fumaric acid**	-	-	-	-	-	-	-	-	-	-	-	-	0.3	0.16	0.11	0.09	-2.33	0.00
**Carboxylic acid**	**L-Lactic acid**	-0.58	0.02	-0.45	0.07	0.41	0.26	0.05	0.65	-0.37	0.04	-0.46	0.09	-0.08	0.84	0.1	0.27	-0.93	0.00
**Carboxylic acid**	**Phthalic acid**	-	-	-1.55	0.00	-0.68	0.47	-0.52	0.65	0.66	0.34	-	-	-	-	-	-	-	-
**Carboxylic acid**	**Pyruvic acid**	-0.69	0.01	-0.99	0.00	-0.64	0.00	1.74	0.45	-0.33	0.95	-0.01	0.10	-0.51	0.01	-0.61	0.00	-1.22	0.00
**Carboxylic acid**	**Succinic acid**	-0.87	0.11	-1.21	0.05	-0.64	0.25	-	-	-	-	-	-	-	-	-	-	-	-
**Carboxylic acid**	**Tetradecanedioic acid**	-0.36	0.10	-0.22	0.28	-0.56	0.04	-	-	-	-	-	-	0.17	0.43	0.12	0.53	0.21	0.11
**Carnitine**	**2-Methylbutyroyl carnitine**	-0.71	0.00	-1.99	0.00	-1.15	0.00	0.06	0.98	-0.36	0.02	-0.9	0.00	-0.3	0.10	-1.16	0.00	-3.31	0.00
**Carnitine**	**Butenylcarnitine**	-	-	1.09	0.29	-0.16	0.60	1.2	0.00	1.88	0.00	2.94	0.01	1.11	0.30	1.5	0.00	1.19	0.01
**Carnitine**	**Decanoylcarnitine**	0.41	0.81	-0.09	0.97	0.22	0.97	0.04	0.90	-1.42	0.31	1.01	0.89	-1.45	0.60	-0.9	0.59	-0.71	0.96
**Carnitine**	**Dodecanedioyl carnitine**	-1.31	0.38	0.61	0.11	0.01	0.95	2.94	0.47	0.86	0.59	-	-	1.22	0.02	1.13	0.04	1.77	0.00
**Carnitine**	**Heptadecanoyl carnitine**	-	-	-1.82	0.37	1.25	0.00	0.24	0.72	-0.97	0.06	-0.38	0.33	0.67	0.68	0.24	0.84	1.2	0.04
**Carnitine**	**L-Carnitine**	-0.17	0.82	0.03	0.54	-0.35	0.11	-0.38	0.40	0.39	0.79	1.69	0.58	1.07	0.05	0.53	0.07	0.98	0.00
**Carnitine**	**L-Hexanoylcarnitine**	0.56	0.55	1.38	0.27	-0.03	1.00	-0.37	0.12	-1.3	0.00	-1.62	0.00	-0.88	0.00	1.75	0.00	1.71	0.00
**Carnitine**	**L-Palmitoylcarnitine**	-8.48	0.19	0.34	0.53	0.47	0.49	0.13	0.86	-0.89	0.13	-0.92	0.11	0.05	0.69	1.24	0.09	1.63	0.03
**Carnitine**	**Pentadecanoyl carnitine**	-1.63	0.15	-0.49	0.14	0.03	0.92	0.72	0.21	-	-	-1.17	0.84	-1.05	0.09	-2.71	0.00	-1.37	0.02
**Carnitine**	**Propionylcarnitine**	-15.38	0.00	-1.42	0.21	-0.65	0.63	0.12	0.85	0.17	0.86	-0.87	0.00	0.14	0.61	-0.89	0.02	-2.79	0.00
**Carnitine**	**Stearoylcarnitine**	-1.15	0.85	2.03	0.01	2.42	0.06	-0.37	0.91	-1.02	0.17	-0.68	0.21	1.09	0.21	1.6	0.03	2.06	0.01
**Carnitine**	**Tiglylcarnitine**	-0.41	0.11	-0.29	0.02	0.22	0.40	0.04	0.87	-0.15	0.07	-0.46	0.04	0.05	0.30	0.09	0.31	-1.88	0.00

**Carnitine**	**trans-2-Dodecenoylcarnitine**	-0.98	0.55	0.81	0.78	-0.53	0.18	-0.57	0.78	-0.35	0.07	0.32	0.33	-0.88	0.02	-0.53	0.07	-0.82	0.00
**Carnitine**	**trans-Hexadec-2-enoyl carnitine**	-	-	-0.74	0.05	0.39	0.93	0.2	0.75	-0.91	0.19	-0.51	0.29	-0.76	0.11	-1.24	0.01	-1.18	0.01
**Ceramide**	**Cer(d18:1/16:0)**	-0.62	0.22	-2.41	0.02	-0.66	0.09	0.42	0.93	-1.65	0.11	-1.28	0.15	-1.47	0.02	-	-	-2.71	0.00
**Ceramide**	**Cer(d18:1/24:1(15Z))**	-2.34	0.14	-2.65	0.02	-3.01	0.07	-	-	-	-	-	-	-1.52	0.12	-3.55	0.01	-3.72	0.01
**Cholesterol ester**	**Cholesteryl acetate**	-1.41	0.62	-0.91	0.04	-0.8	0.18	-0.09	0.16	-0.4	0.10	-1.31	0.00	-	-	-	-	-	-
**Fatty acid**	**Glycerol 3-phosphate**	-1	0.00	-1.57	0.00	-0.66	0.01	-0.56	0.11	-0.79	0.14	-2	0.00	-0.11	0.61	0.02	0.93	-0.77	0.02
**Fatty acid**	**Linoleamide**	-2.79	0.12	-1.52	0.63	-0.07	0.97	-0.06	0.56	-3.04	0.04	0.04	0.68	-1.17	0.38	-0.8	0.36	-1.23	0.29
**Fatty acid**	**Stearic acid**	-0.4	0.47	0.43	0.44	-0.27	0.59	0.24	0.76	0.72	0.80	0.74	0.72	0.25	0.45	0.31	0.10	0.74	0.00
**Fatty acid**	**Stearoylethanolamide**	-	-	-	-	-	-	-	-	-1.21	0.03	-1.25	0.03	-0.78	0.59	-1.33	0.17	-1.38	0.13
**Fatty acid**	**Tetraglyme**	-	-	0.72	0.11	-2.02	0.04	0.82	0.39	0.68	0.82	2.35	0.18	0.73	0.08	0.38	0.01	0.89	0.00
**Fatty amide**	**Oleamide**	-2.79	0.12	-1.52	0.63	-0.07	0.97	-0.06	0.56	-3.04	0.04	0.04	0.68	-1.17	0.38	-0.8	0.36	-1.23	0.29
**Furanone**	**Ascorbic acid**	-0.03	0.96	-0.83	0.03	-0.21	0.24	1.34	0.49	-	-	1.43	0.34	-2.16	0.00	-1.55	0.00	-0.25	0.09
**Inorganic compound**	**Pyrophosphate**	-0.04	0.29	0.93	0.01	0.48	0.19	0.55	0.31	0.63	0.15	0.94	0.02	0.88	0.07	1.51	0.01	1.67	0.00
**Keto acid**	**Acetoacetic acid**	7.66	0.01	-0.48	0.00	-0.04	0.23	-0.07	0.39	-0.21	0.05	-0.69	0.00	-0.17	0.34	-0.42	0.02	-1.47	0.00
**Keto acid**	**alpha-Ketoglutaric acid**	-0.39	0.01	-1.35	0.00	0.35	0.01	0.49	0.01	0.12	0.20	-0.3	0.13	-0.64	0.00	-0.35	0.04	-0.62	0.01
**Keto acid**	**Levulinic acid**	-	-	-	-	-	-	-	-	-1.14	0.01	-0.57	0.03	-1.08	0.09	-0.77	0.01	-2.36	0.00
**Nucleobase**	**Adenine**	-1.24	0.00	-1.92	0.00	-0.76	0.01	-	-	-	-	-	-	-0.27	0.16	-1.61	0.00	-5.17	0.00
**Nucleoside**	**2'-Deoxycytidine**	-	-	-	-	-	-	-	-	-	-	-	-	-0.4	0.54	-0.09	0.65	-0.9	0.00
**Nucleoside**	**5'-Methylthioadenosine**	-0.95	0.21	-1.43	0.00	-0.76	0.00	-1.54	0.19	-0.39	0.58	-0.79	0.83	-0.56	0.05	-0.73	0.00	-0.89	0.00

**Nucleoside**	**Adenosine**	-0.95	*0.21*	0.86	*0.01*	1.1	*0.12*	-1.54	*0.19*	-0.39	*0.58*	-0.79	*0.83*	-0.56	0.05	-0.73	0.00	-0.89	0.00
**Nucleoside**	**Thymidine**	-	-	-	-	-	-	-	-	-	-	-	-	-0.25	0.21	0.17	0.88	-2.14	0.03
**Nucleotide**	**3'-AMP**	-	-	0.86	*0.01*	1.1	*0.12*	-	-	-	-	-	-	-	-	-	-	0.44	0.94
**Nucleotide**	**CDP-ethanolamine**	-	-	-	-	-	-	-	-	-	-	-	-	2.51	0.00	-	-	2.85	0.00
**Nucleotide**	**Cytidine 5'-diphosphocholine**	-	-	-	-	-	-	-	-	-	-	-	-	1.41	0.00	1.67	0.00	1.25	0.01
**Nucleotide**	**NAD**	4.71	*0.27*	-1.48	*0.00*	-0.55	*0.04*	0.54	*0.24*	-0.03	*0.18*	-0.45	*0.01*	-0.27	0.16	-0.59	0.00	-0.95	0.00
**Nucleotide**	**NADH**	-0.5	*0.02*	-1.18	*0.00*	-0.48	*0.05*	-	-	-	-	-4.74	*0.00*	-	-	-	-	-	-
**Nucleotide**	**Uridine**	-0.82	*0.02*	-1.87	*0.00*	-1.03	*0.01*	0.42	*0.36*	-1.05	*0.43*	-1.99	*0.01*	-0.64	0.05	-0.71	0.02	-1.01	0.00
**Nucleotide**	**Uridine 5'-diphosphogalactose**	-	-	-	-	-	-	-0.05	*0.24*	-0.22	*0.07*	-0.84	*0.01*	-	-	-	-	-	-
**Nucleotide**	**Uridine 5'-diphosphoglucuronic acid**	-0.6	*0.00*	-1.41	*0.00*	-0.95	*0.00*	0.11	*0.77*	-	-	-	-	-0.54	0.02	-0.9	0.00	-1.94	0.00
**Nucleotide**	**Uridine diphosphate-N-acetylglucosamine**	-0.49	*0.03*	-1.99	*0.00*	-1.37	*0.00*	-0.86	*0.04*	-0.66	*0.03*	-1.69	*0.00*	-0.51	0.03	-1.03	0.00	-2.03	0.00
**Nucleotide**	**Uridine diphosphategalactose**	-0.91	*0.02*	-1.44	*0.00*	-0.58	*0.01*	-	-	-	-	-	-	-0.72	0.02	-1.13	0.00	-2.66	0.00
**PC**	**1,2-Dipalmitoleoyl-sn-glycero-3-phosphocholine**	-	-	-3.11	*0.01*	-2.79	*0.03*	0.47	*0.25*	-1.75	*0.13*	-1.31	*0.06*	-	-	-	-	-	-
**PC**	**LysoPC(14:1(9Z)/0:0)**	-1.74	*0.58*	-3.65	*0.00*	-2.42	*0.01*	-	-	-	-	-	-	-	-	-	-	-	-
**PC**	**LysoPC(24:1(15Z))**	-2.41	*0.53*	-2.05	*0.04*	-1.76	*0.17*	0.75	*0.30*	-1.42	*0.22*	-2.09	*0.05*	-2.55	0.03	-4.21	0.00	-3.98	0.01
**PC**	**LysoPC(P-16:0/0:0)**	-1.87	*0.31*	-0.97	*0.11*	-1.33	*0.20*	-	-	-	-	-	-	-1.88	0.05	-3.34	0.01	-3.25	0.01
**PC**	**PC(16:0/18:1(9Z))**	-	-	-0.82	*0.22*	-0.68	*0.76*	0.42	*0.27*	-1.48	*0.26*	-1.24	*0.05*	-0.62	0.13	-2.79	0.00	-2.88	0.00
**PC**	**PC(16:0/18:3 (9Z,12Z,15Z))**	-4.22	*0.31*	-0.46	*0.11*	-1.24	*0.12*	0.45	*0.24*	-	-	-	-	-0.48	0.33	-1.25	0.01	-1.52	0.03

PC	PC(18:1(9Z)e/2:0)	-	-	-0.49	0.14	-1.53	0.14	0.52	0.49	-1.36	0.14	-1.86	0.01	-2.8	0.03	-3.98	0.00	-3.3	0.01
PE	1-oleoyl-2-linoleyl-sn-glycero-3-phosphoethanolamine	-0.81	0.07	-2.51	0.00	-1.81	0.00	-	-	-	-	-	-	-0.58	0.27	-1.57	0.02	-3.21	0.00
PE	1-Palmitoyl-2-linoleoyl PE	-0.81	0.07	-2.65	0.00	-1.61	0.01	0.02	0.85	-0.88	0.26	-0.29	0.46	-	-	-	-	-	-
PE	LysoPE(18:0/0:0)	-	-	-1.93	0.34	-1.02	0.37	-	-	-	-	-1.17	0.15	-2.13	0.03	-2.82	0.00	-1.44	0.06
PE	LysoPE(18:1(9Z)/0:0)	-1.23	0.01	-2.06	0.00	-1.09	0.00	0.79	0.12	-	-	-	-	-	-	-	-	-	-
PE	PE(16:0/22:6 (4Z,7Z,10Z,13Z,16Z,19Z))	-0.05	0.72	-0.68	0.01	-0.12	0.28	-	-	-1.27	0.55	-	-	-	-	-	-	-	-
PE	PE(18:0/20:4 (5Z,8Z,11Z,14Z))	-	-	-0.1	0.14	0.15	0.13	1.43	0.32	-0.51	0.40	0.17	0.62	2.08	0.40	1.74	0.72	1.64	0.41
PE	PE(P-16:0/20:4 (5Z,8Z,11Z,14Z))	0.51	0.72	0.04	0.47	0.6	0.48	-	-	-0.34	0.15	-	-	-	-	-	-	-	-
Peptide	Carnosine	5.16	0.00	-0.22	0.42	0.04	0.78	-0.15	0.27	-0.44	0.01	-0.36	0.03	-0.93	0.04	-1.16	0.00	-1.53	0.00
Peptide	L-Glutathione (reduced)	-1.02	0.00	-3.26	0.00	-1.35	0.00	-0.04	0.37	-0.29	0.10	-1.65	0.01	-0.63	0.01	-1.24	0.00	-1.72	0.00
Peptide	L-Glutathione (oxidized)	-	-	1.48	0.01	-	-	1.24	0.12	-	-	-	-	-0.17	0.56	-	-	-	-
Peptide	Ophthalmic acid	5.85	0.08	-2.18	0.00	0.2	0.36	0.38	0.00	0.52	0.00	0.82	0.01	1.59	0.00	1.62	0.00	1.47	0.00
Peptide	Pro-leu	8.41	0.00	-1.03	0.00	0.1	0.97	0.97	0.00	1.22	0.00	1.33	0.00	-0.22	0.73	-0.11	0.69	-0.45	0.00
Phenylketone	Kynurenine	-1.65	0.27	-	-	-	-	-	-	-0.28	0.44	1.24	0.08	-	-	-	-	-	-
Polyamine	N-Acetylputrescine	-1.25	0.00	-2.83	0.00	-2.4	0.00	-	-	-	-	-	-	-	-	-	-	-	-
Polyamine	N1,N12-Diacetyl spermine	1.84	0.40	3.11	0.00	5.71	0.00	3	0.00	3.68	0.00	4.5	0.00	4.79	0.00	4.4	0.00	5.44	0.00
Polyamine	N1,N8-Diacetyl spermidine	1.03	0.00	0.5	0.00	2.15	0.00	1.13	0.00	1.37	0.00	0.71	0.01	1.27	0.00	0.53	0.00	0.5	0.01
Polyamine	N8-Acetyl spermidine	8.44	0.02	0.73	0.00	2.71	0.00	1.01	0.32	1.22	0.00	0.77	0.00	1.03	0.00	0.62	0.00	0.04	0.80

**PUFA**	**Alpha-Linolenic acid**	-	-	0.61	*0.93*	1.98	*0.37*	-0.29	*0.60*	-1.03	*0.83*	0.31	*0.62*	-1.26	0.52	-1.34	0.59	-1.47	0.52
**PUFA**	**Linolenelaidic acid**	-	-	2.32	*0.96*	-1.85	*0.02*	0.05	*0.53*	-1.74	*0.17*	1.01	*0.26*	-1.36	0.59	-2.73	0.18	0.01	0.52
**Pyridine**	**Pyridoxal**	-	-	0.82	*0.10*	0.24	*0.93*	1.31	*0.53*	1.01	*0.71*	1.04	*0.63*	0.19	0.62	0.22	0.41	0.23	0.76
**Pyridine**	**Pyridoxamine**	-1.36	*0.17*	-1.96	*0.01*	-0.32	*0.82*	1.79	*0.38*	0.01	*0.80*	0.51	*0.67*	-0.09	0.44	0.06	0.69	-0.48	0.22
**Pyridine**	**Pyridoxine**	-0.74	*0.01*	-1.5	*0.00*	6.99	*0.00*	-	-	-	-	-1.13	*0.66*	0.6	0.85	1.39	0.04	-	-
**Pyrrolidine**	**1-Methyl pyrrolidine**	-	-	2.06	*0.02*	0.92	*0.08*	-0.25	*0.23*	0.91	*0.63*	0.16	*0.90*	0.88	0.01	1.01	0.01	1.54	0.00
**Pyrrolidine**	**Pyrrolidine**	-0.6	*0.34*	-0.89	*0.55*	-0.82	*0.33*	-0.99	*0.65*	-2.59	*0.09*	1.87	*0.50*	-2.09	0.04	-0.07	0.55	-0.25	0.22
**SM**	**SM(d18:0/14:0)**	-3.11	*0.13*	-2.31	*0.04*	-3.1	*0.10*	-	-	-	-	-	-	-2.34	0.02	-3.86	0.00	-4.08	0.00
**SM**	**SM(d18:1/16:0)**	-4.02	*0.09*	-5.16	*0.01*	-3.9	*0.04*	1.05	*0.27*	-1.27	*0.29*	-1.2	*0.09*	-2.31	0.04	-3.37	0.00	-4.28	0.00

Class, name, Log_2 fold change, and the p-value (p) is represented for each compound. PC: phosphocholine; PE: phosphoethanolamine; PUFA: poly unsaturated fatty acid; SM: Sfingomyelin

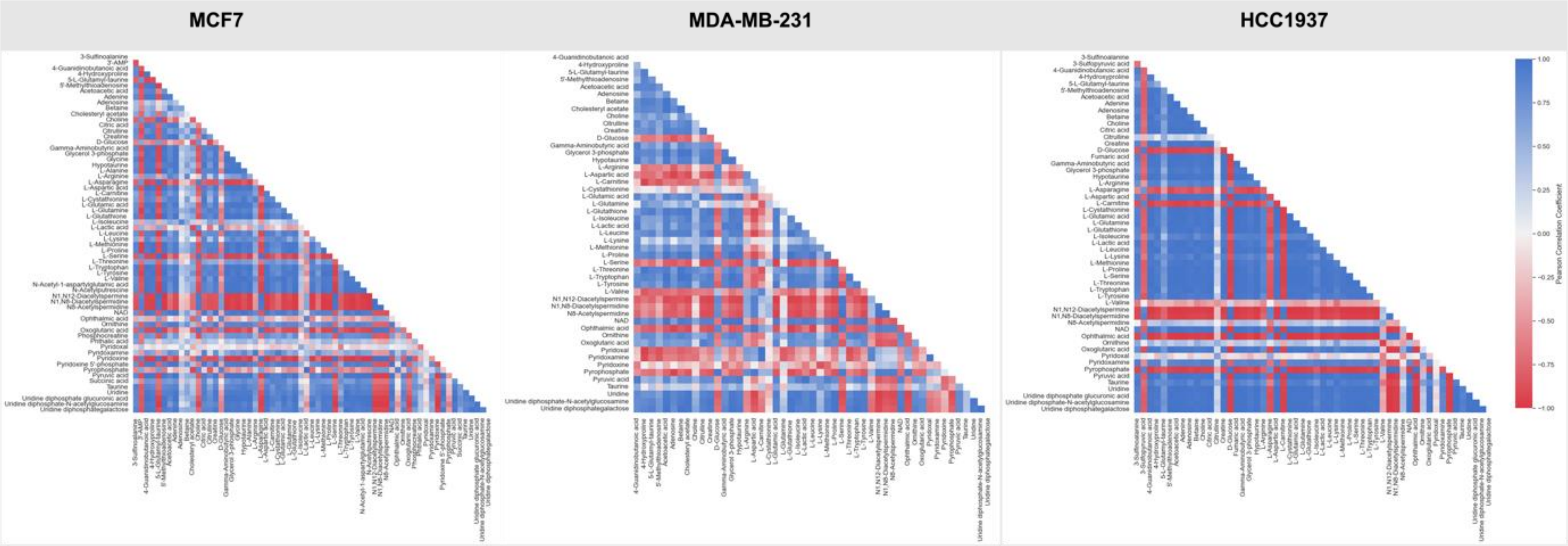

Figure S8. Pearson's correlation analysis between the relevant metabolites identified within each different breast cancer cell line. Pearson's coefficient is set in a range of 1 to -1, indicative of a positive and negative correlation, respectively.

Table S8. Effect scores of enriched metabolic genes in MCF-7, MDA-MB-231 and HCC1937 cells evaluated through the Dependency Map Portal (DepMap) database. Fitness effect score is based on the Chronos algorithm.

PATHWAY			MCF7	MDA-MB-231	HCC1937
	FULL NAME	***GENE***	***FITNESS EFFECT SCORE***	***FITNESS EFFECT SCORE***	***FITNESS EFFECT SCORE***
**Arginine biosynthesis**					
	Glutamine synthetase	GLUL	-0.125358532	-0.054074159	-0.019578279
	Glutaminase 2	GLS2	-0.048640596	-0.10856474	-0.052528908
	Glutamic-oxaloacetic transaminase 1	GOT1	0.028242354	-0.063893637	-0.073798616
	Glutamic--Pyruvic Transaminase	GPT	0.037215894	0.10892219	-0.216893048
	Ornithine transcarbamylase	OTC	0.147486186	0.100967984	0.138874274
	Arginase 1	ARG1	0.227985584	0.091116773	0.024848929
	Nitric Oxide Synthase 1	NOS1	-0.023334093	-0.137541143	-0.002959303
	Argininosuccinate Synthase 1	ASS1	-0.184888377	-0.402563868	-0.562629354
	Argininosuccinate Lyase	ASL	-0.113055241	-0.123584385	-0.047769185
**Alanine, aspartate and glutamate metabolism**					
	Succinate-semialdehyde dehydrogenase	SSDH	-0.149665574	-0.092021306	-0.23551055
	Aminobutyrate aminotransferase	ABAT	-0.047346955	-0.075226835	0.005737048
	Glutamate Decarboxylase 1	GAD1	-0.044126537	0.003160126	-0.09859572
	Glutamate dehydrogenase 1	GLUD1	-0.021542034	-0.072836976	0.080316519
	Asparagine Synthetase	ASNS	-0.374287356	-0.55876465	-0.403428363
	Glycine Amidinotransferase	GATM	-0.046046866	-0.119724747	-0.183393939
**Arginine and proline metabolism**					
	Ornithine Decarboxylase 1	ODC1	-0.05327327	0.048019018	0.109983109
	Spermidine/Spermine N1-Acetyltransferase	SAT2	-0.19112135	0.004246237	-0.175388686
**Taurine and hypotaurine**					
	Glutamate Decarboxylase 1	GAD1	-0.044126537	0.003160126	-0.09859572
	Glutamate Decarboxylase Like 1	GADL1	0.080229488	-0.115932344	0.053750068
	Gamma-Glutamyltransferase 6	GGT6	-0.048655115	-0.089863757	-0.007089641
**Glycine, serine and threonine metabolism**					
	Serine Dehydratase	SDS	0.2145656	0.012084165	0.021749979
	Serine hydroxymethyltransferase	SHMT	0.089762197	-0.106194689	-0.173416165
	Alanine-Glyoxylate Aminotransferase	AGXT	-0.000518467	0.056342348	-0.085768781
	Guanidinoacetate methyltransferase	GAMT	-0.067677445	0.015662028	0.04233071
	Antiquitin 1	ATQ1	-0.154061124	-0.078707501	0.024371521
	L-Arginine:glycine amidinotransferase	AGAT	-0.046046866	-0.119724747	-0.183393939
**Vitamin B6 metabolism**					
	Pyridoxine 5-prime-phosphate oxidase	PNPO	0.002519954	-0.063587768	0.041033014
**Cysteine and methionine metabolism**					
	Betaine-homocysteine methyltransferase	BHMT	-0.101017733	-0.080660636	0.044393697
	Glutathione Synthetase	GSS	-0.095373599	-0.009352038	0.168192909
	ChaC Glutathione Specific Gamma-Glutamylcyclotransferase 1	CHAC	0.103413191	-0.118655017	-0.185636384
